# Full-Length Recombinant hSP-D Binds and Inhibits SARS-CoV-2

**DOI:** 10.3390/biom11081114

**Published:** 2021-07-28

**Authors:** Raquel Arroyo, Shawn N. Grant, Miriam Colombo, Lucia Salvioni, Fabio Corsi, Marta Truffi, Davide Ottolina, Brett Hurst, Marc Salzberg, Davide Prosperi, Paul S. Kingma

**Affiliations:** 1 Division of Neonatology and Pulmonary Biology, Cincinnati Children’s Hospital, Cincinnati, OH 45229, USA; raquel.arroyo@cchmc.org; 2 Airway Therapeutics Inc., Cincinnati, OH 45249, USA; grant@airwaytherapeutics.com (S.N.G.); salzberg@airwaytherapeutics.com (M.S.); 3 NanoBio laboratory, Department of Biotechnology and Bioscience, University of Milano-Bicocca, 20126 Milano, Italy; miriam.colombo@unimib.it (M.C.); lucia.salvioni@unimib.it (L.S.); davide.prosperi@unimib.it (D.P.); 4 Istituti Clinici Scientifici Maugeri IRCCS, via Maugeri 4, 27100 Pavia, Italy; fabio.corsi@icsmaugeri.it (F.C.); marta.truffi@icsmaugeri.it (M.T.); 5 Division of Anesthesiology and Intensive Care Medicine, ASST Fatebenefratelli Sacco, Luigi Sacco Hospital, University of Milan, 20157 Milano, Italy; ottolina.davide@asst-fbf-sacco.it; 6 Institute for Antiviral Research, Utah State University, Logan, UT 84322, USA; brett.hurst@usu.edu; 7 Department of Pediatrics, University of Cincinnati, Cincinnati, OH 45229, USA

**Keywords:** SP-D, COVID-19, spike-protein, ACE2, anti-inflammatory

## Abstract

SARS-CoV-2 infection of host cells is driven by binding of the SARS-CoV-2 spike-(S)-protein to lung type II pneumocytes, followed by virus replication. Surfactant protein SP-D, member of the front-line immune defense of the lungs, binds glycosylated structures on invading pathogens such as viruses to induce their clearance from the lungs. The objective of this study is to measure the pulmonary SP-D levels in COVID-19 patients and demonstrate the activity of SP-D against SARS-CoV-2, opening the possibility of using SP-D as potential therapy for COVID-19 patients. Pulmonary SP-D concentrations were measured in bronchoalveolar lavage samples from patients with corona virus disease 2019 (COVID-19) by anti-SP-D ELISA. Binding assays were performed by ELISAs. Protein bridge and aggregation assays were performed by gel electrophoresis followed by silver staining and band densitometry. Viral replication was evaluated in vitro using epithelial Caco-2 cells. Results indicate that COVID-19 patients (*n* = 12) show decreased pulmonary levels of SP-D (median = 68.9 ng/mL) when compared to levels reported for healthy controls in literature. Binding assays demonstrate that SP-D binds the SARS-CoV-2 glycosylated spike-(S)-protein of different emerging clinical variants. Binding induces the formation of protein bridges, the critical step of viral aggregation to facilitate its clearance. SP-D inhibits SARS-CoV-2 replication in Caco-2 cells (EC_90_ = 3.7 μg/mL). Therefore, SP-D recognizes and binds to the spike-(S)-protein of SARS-CoV-2 in vitro, initiates the aggregation, and inhibits viral replication in cells. Combined with the low levels of SP-D observed in COVID-19 patients, these results suggest that SP-D is important in the immune response to SARS-CoV-2 and that rhSP-D supplementation has the potential to be a novel class of anti-viral that will target SARS-CoV-2 infection.

## 1. Introduction

Coronavirus Disease 2019 (COVID-19) is a complex pneumonia caused by SARS-CoV-2, an enveloped RNA virus, whose surface is decorated with a glycosylated spike-(S)-protein [1,2]. The subunit-S_1_ of the spike-protein interacts with the human angiotensin-converting-enzyme-2 (ACE2) receptor in type II pneumocytes [2,3,4]. The virus is internalized by the host cells, resulting in viral replication [4]. New copies of SARS-CoV-2 are externalized to infect more cells, increasing the viral load in lungs, exacerbating the pro-inflammatory response, and extending the cellular and epithelial lung damage [5]. In severe cases of COVID-19, pneumonia progresses to complex ALI/ARDS, respiratory failure, septic shock, and even death [5,6,7]. To date, remdesivir has shown effect by shortening the recovery time of patients 4 days [8], and dexamethasone has reduced the mortality of critical patients by 33% [9]. However, treatments that specifically target the virus and the exacerbated inflammatory response with higher efficacy are still needed.

Some new variants of SARS-CoV-2 have emerged due to the mutation of certain amino acids in the viral sequence, some of them located in the spike protein. The B.1.1.7. (so-called U.K. variant), B.1.351 (South Africa) and P.1 (Brazil) are some of the most concerning ones currently due to their spread around the world and/or resulting clinical disease severity [10,11,12]. These variants enclose different mutations, but, the three of them share two common mutations in the S_1_-protein: N501Y and D614G [13,14].

Surfactant protein SP-D is a collectin protein with an important role in the front-line innate immune defense of the lungs [15,16]. SP-D binds to glycosylated ligands on pathogens and triggers opsonization, aggregation, and direct killing of microbes, which facilitates their clearance from the lungs by phagocytic cells such as macrophages [15,16]. SP-D dodecamers and higher order oligomers have shown an increased activity and potency in this anti-microbial function [17]. In addition to critical roles in pathogen clearance, SP-D has also shown an anti-inflammatory effect in respiratory infections as well as in lung injury induced by mechanical ventilation. SP-D decreases the levels of pro-inflammatory cytokines, the neutrophilic response, NETosis, and the resulting lung tissue damage [16,18,19,20,21,22,23]. Animal models have consistently demonstrated an association between higher levels of pulmonary SP-D and improved outcomes following viral, bacterial, or mechanical lung injury. Likewise, human studies have demonstrated lower mortality rates in ARDS patients with high levels of pulmonary SP-D [24]. Full length recombinant hSP-D has been successfully produced in mammalian cells, showing comparable structure and activity to human native SP-D [25,26,27]. Therefore, we investigated the binding and inhibitory activity of SP-D against SARS-CoV-2 and the potential of rhSP-D as a novel class of antiviral therapeutic for COVID-19.

## 2. Material and Methods

### 2.1. Materials

Full length recombinant human rhSP-D was produced in a human cell line GlycoExpress^®^ (GEX) developed in Glycotope-GmbH. The rhSP-D variant is Met^11^, Thr^160^, Ser^260^. The purification process followed has been described elsewhere [19,26].

Recombinant SARS-CoV-2 spike protein variants (S_1_-subunit) and recombinant human ACE2 protein were expressed in HEK293 cells and purchased from SinoBiologicals (#40591-V08H, #40591-V05H1, #10108-H05H, #40591-V08H3, #40591-V08H10), AcroBiosystems (#S1N-C52H3, #S1N-C52Hk, #S1N-C52Hg, #S1N-C52Hn, #S1N-C52Hp, #S1N-C52Hm), The NativeAntigen Company (#REC31806-100-HRP) and from Biomart Creative (#ACE2-736H).

### 2.2. Determination of hSP-D Levels in Bronchoalveolar Lavage of COVID-19 Patients

Bronchoscopy and bronchoalveolar lavage fluid (BALF) processing were conducted following the protocol described elsewhere [28]. Bronchoscopies were performed in sedated, paralyzed, and mechanically ventilated adult (≥18 years old) patients (*n* = 12) with confirmed COVID-19 by PCR test admitted to hospital between March and April of 2020; more details about when the bronchoscopy was performed for each patient in relation to hospitalization and intubation date are provided in Table 1.

BALF aliquots were collected after 5–6 bolus of 20 mL sterile saline, the initial 20 mL were discarded. The suspensions were centrifuged at 400× *g* for 10 min to remove cells and supernatants were saved and inactivated with 0.2% SDS, 0.1% Tween20 followed by 15 min at 65 °C. The resulting BALF were stored at −20 °C until analysis. SP-D levels were quantified by human anti-SP-D ELISA (Biovendor). BALF were collected after authorization by the Ethic Committee of Ospedale Luigi Sacco (experimentation number 2020/ST/145).

### 2.3. SARS-CoV-2 Spike-Protein and rhSP-D ELISA Binding Assay

Microtiter plates were coated with the desired variant of recombinant S_1_-spike-protein (0.4 μg in 200 μL/well). Washes and dilutions were performed with 0.05% TBS-tween, 5 mM CaCl_2_. Wells were blocked with 2%-BSA and serial diluted rhSP-D (10 μg/mL to 9.8 ng/mL) was added to the wells. Bound rhSP-D was detected with a mouse anti-SP-D antibody (Seven Hills Bioreagents), followed by an anti-mouse IgG horseradish peroxidase (HRP)-conjugated antibody (Cell Signaling). The plates were developed with TMB (Surmodics) for 10 min and the reaction was stopped with 2N H_2_SO_4_. Plates were read for absorption at 450 nm. Non-binding negative controls were included, using 50 mM EDTA to prevent calcium-dependent binding or 200 mM maltose, also with 5 mM calcium, to create binding competition between maltose and S_1_-protein. To address nonspecific binding to the plate, wells were coated with 1% BSA instead of S_1_-protein.

Analysis of the binding isotherms was performed with GraphPad-Prism 8, considering total binding and one site, to determine the apparent dissociation constant (kd). In the new variants, the binding was represented as % relative absorbance, to allow averaging of the absorbance signal of experimental replicates performed on different days. The O.D. signal of rhSP-D at maximum concentration tested (10 μg/mL), bound to the Wuhan variant, was considered as 100%. All experiments were performed at least twice on different days.

### 2.4. Protein-Bridge (Aggregation) Assay

#### 2.4.1. rhSP-D First Approach

RhSP-D (2 or 4 μg) was incubated 30 min at room temperature with maltose-coated agarose beads in 50 μL TBS (150 mM NaCl, 20 mM Tris (pH 7.4))-10 mM CaCl_2_ buffer. The supernatant (S_A_) with the excess unbound rhSP-D was separated by centrifugation and saved. The beads were washed with TBS-CaCl_2_. Then, 2 μg of S_1_-protein or buffer (negative control) were added to the beads and the final volume was adjusted to 50 μL with TBS-CaCl_2,_ or with TBS-EDTA 20 mM in the non-binding control. After incubation (2 h at room temperature), the beads were centrifuged and the supernatant (S_B_) was saved. The beads (pellet) were washed with the appropriate buffer followed by elution of the bound rhSP-D with TBS-EDTA 20 mM, the eluted fraction from the pellet (P) was saved for analysis.

#### 2.4.2. Pre-Mix Approach

RhSP-D (2 or 4 μg) and S_1_-protein (2 μg) were pre-mixed and incubated for 2 h to favor binding and aggregation of S_1_-protein by rhSP-D. Then, the mix was added to the beads. After incubation (30 min, room temperature), beads were centrifuged and the supernatant (S_A_) was saved. The beads were washed and eluted as previously described, saving the eluted fraction (P) for analysis.

In both methods, the presence of rhSP-D and S_1_-protein in fractions (S_A_, S_B_ and P) were determined by SDS-PAGE under reducing conditions and developed by silver staining. Intensity of rhSP-D bands from the samples that contained 4 μg of rhSP-D was quantified by densitometry, in duplicate, with ImageJ software. The relative intensity of the rhSP-D band in the pellet fraction (P) was calculated considering 100% to be the intensity of the “S_A_” band in the buffer control at 5 mM calcium.

### 2.5. Competition of SARS-CoV-2 Spike-Protein Binding to ACE2 Protein by rhSP-D

#### 2.5.1. Binding of ACE2 to S_1_-Protein in the Presence of rhSP-D

Plates were coated with purified S_1_-spike-protein (Wuhan variant). RhSP-D (0.1 to 1 μg/mL) in TBS-Ca 5 mM or buffer (negative control) were added to the wells and incubated for 2 h. Without washing, human ACE2 protein (0.186 to 1.5 μg/mL) was added to the wells at each of the rhSP-D concentrations, a control with TBS buffer instead of ACE2 was also included. After incubation (30 min), bound ACE2-mFc was detected with an anti-mouse IgG HRP-conjugated antibody (Cell Signaling); plates were developed as previously described.

#### 2.5.2. Binding of S_1_-Protein to rhSP-D in the Presence of ACE2

Plates were coated with rhSP-D (5 μL/mL, 200 μL/well). S_1_-protein-HRP-tagged at different concentrations or buffer (negative control), were added to the wells and incubated for 2 h. Without washing, human ACE2 protein His-tagged was added to the wells to reach 3, 0.375, or 0.045 μg/mL at each of the S_1_-protein concentrations. After incubation (30 min), bound S_1_-protein-HRP was detected directly with TMB and the reaction was stopped with 2N H_2_SO_4_.

### 2.6. Inhibition of Viral Replication: Reduction of Virus Yield (VYR) Assay

Monolayers utilizing the human Caco-2 cell line were prepared 24 h prior to virus infection in 96-well microplates at 37 °C with 5% CO_2_. Growth media was removed from the cells and the rhSP-D was applied and tested in triplicate at eight serial half-log_10_ dilution concentrations, starting at 100 μg/mL. SARS-CoV-2 (strain USA/WA_1/2020_) at 200 CCID_50_ (50% cell culture infectious dose) was added to wells designated for virus infection; MOI = 0.02. Controls were performed with uninfected and untreated cells (cell controls) and also with infected and not rhSP-D-treated cells, using an identical control buffer lacking rhSP-D (virus controls). Plates were incubated at 37 °C for 72 h. A sample of the supernatant was taken for *n* = 3 replicates from each infected well for testing and virus titer determination. Titration of the viral samples previous collected is performed by endpoint dilution as described elsewhere [29]. Serial dilutions of virus were made and plated into wells containing fresh cell monolayers of Vero 76 cells. Plates were incubated, and cells were scored for presence or absence of virus after a distinct cytopathogenic effect was observed, and the CCID_50_ calculated using the Reed–Muench method [29]. The 90% (one log_10_) effective concentration (EC_90_) was calculated.

Cell toxicity of rhSP-D was evaluated in additional plate wells by using a neutral red dye that penetrates into living cells and allows quantification of viable cells (the more intense the red color, the larger the number of viable cells present in the wells). The dye content in each well was quantified using a spectrophotometer at 540 nm wavelength.

### 2.7. Statistics Analysis

Statistical analyses were performed on GraphPad-Prism 8. ANOVA, Wilcoxon, Friedman, or Kruskal–Wallis with Dunn’s post-hoc test were performed where indicated. Significant differences were considered with a *p*-value of <0.05 (*).

## 3. Results

### 3.1. COVID-19 Patients Show Low Concentrations of Pulmonary SP-D

SP-D levels have been screened in the BALF of several respiratory diseases that exhibit acute lung injury [30]. BALF samples were collected from COVID-19 patients with different age, characteristics, and comorbidities (if present), which are indicated in Table 1. The pulmonary levels of SP-D in COVID-19 patients showed a median concentration of 68.9 ng/mL (mean = 244.8 ng/mL, *n* = 12).

### 3.2. Recombinant hSP-D Binds to the S-Protein of SARS-CoV-2

**Figure 1:**
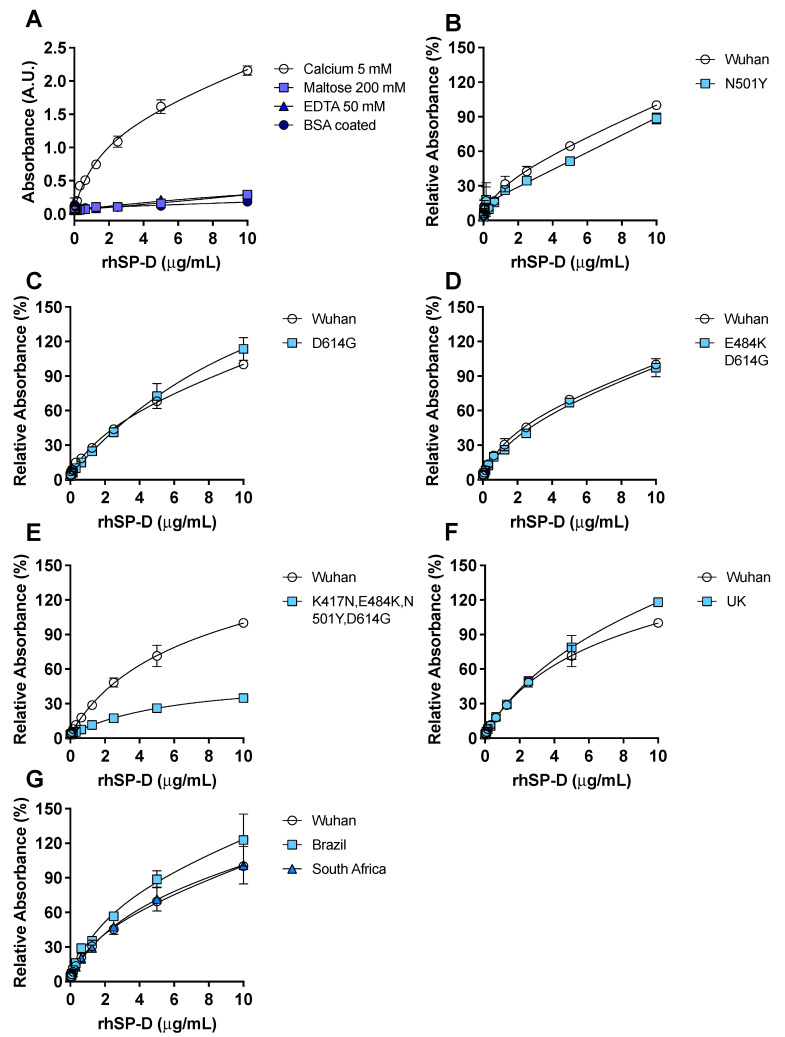
rhSP-D binds to the spike-(S)-protein of SARS-CoV-2. ELISA binding assays were performed, coating the plate wells with S_1_-protein. (**A**) The binding of rhSP-D to the Wuhan variant S_1_-protein was determined by anti-SP-D ELISA under different conditions: 5 mM calcium, 200 mM maltose, and 50 mM EDTA; additionally, wells were coated with bovine serum albumin (BSA) instead of S-protein to determine nonspecific binding of rhSP-D and show that the binding to the S-protein was specific. The binding of rhSP-D to S_1_-protein in the presence of calcium was significantly different to the binding of rhSP-D in the presence of maltose (*p* = 0.004) or EDTA (*p* = 0.02) (Kruskal–Wallis with Dunn’s post hoc test). Applying a one-binding site model the apparent Kd was 1.65 and the apparent B_max_ was 1.35. Binding of rhSP-D to the S_1_-protein variant from Wuhan, compared to a S_1_-protein enclosing different mutations, was tested: N501Y (**B**), D614G (**C**), E484K + D614G (**D**) or K417N + E484K + N501Y + D614G (**E**); binding was significantly different in the last mutant compared to the Wuhan variant (**E**) *p* = 0.0005 (Wilcoxon test). (**F**,**G**) rhSP-D binding to relevant clinical variants of S_1_-protein (Wuhan, Brazil (**G**), South Africa (**G**) and U.K. (**F**)) was determined and rhSP-D bound to all the variants tested; binding was significantly different in the Brazil variant compared to the Wuhan variant (**G**) *p* = 0.002 (Friedman with Dunn’s test). *n* = 4, error bars represent standard deviation.

Binding experiments indicated that rhSP-D recognized and bound to the subunit S_1_ of the spike protein from the first identified variant of SARS-CoV-2 (Wuhan variant) (Figure 1A) with a similar apparent dissociation constant when rhSP-D (Kd = 1.65) (Figure 1A) or S_1_-protein (Kd = 2.02) (Figure S1) was the ligand.

Binding of rhSP-D to S_1_-protein was inhibited by EDTA confirming that it is calcium-dependent. Binding competition with maltose, which also binds to the CRD of rhSP-D in a calcium-dependent manner, abrogated the binding of rhSP-D to S-protein. The binding of rhSP-D to S_1_-protein in the presence of calcium was significantly different (*p* < 0.05) to the binding with EDTA or maltose. Strongly suggesting that the CRD of rhSP-D mediates the binding to the carbohydrates described on the S_1_-protein of SARS-CoV-2 [2,31]. Binding of rhSP-D to the S_1_-protein bearing the mutations identified in the U.K. B.1.1.7. variant (HV69-70, N501Y, D614G), in the South African B.1.351 lineage (L18F, D80A, D215G, R246I, K417N, E484K, N501Y, D614G), or in the Brazil P.1 lineage (L18F, T20N, P26S, D138Y, R190S, K417T, E484K, N501Y, D614G, H655Y) was tested. rhSP-D binding to the S_1_-protein from the U.K. (Figure 1F), Brazil and South Africa (Figure 1G) physiologically relevant variants was similar to the Wuhan variant; it showed a slightly higher affinity for the Brazilian one (*p* = 0.002). The significance for rhSP-D binding of specific amino acid mutations found in the new variants was tested. N501Y and D614G were addressed individually since they are common to all physiologically relevant variants. The mutation N501Y decreased rhSP-D binding when compared to the Wuhan original variant (Figure 1B), on the other hand the D614G (Figure C) and E484K + D614G (Figure 1D) had almost no effect in rhSP-D binding to the spike protein when compared to the Wuhan variant. However, the binding was significantly decreased (*p* = 0.0005) with the combination K417N, E484K, N504Y, D614G (Figure 1E).

The following experiments were performed with the S_1_-protein from the Wuhan variant.

### 3.3. rhSP-D Forms Protein Bridges with the S-Protein of SARS-CoV-2

**Figure 2:**
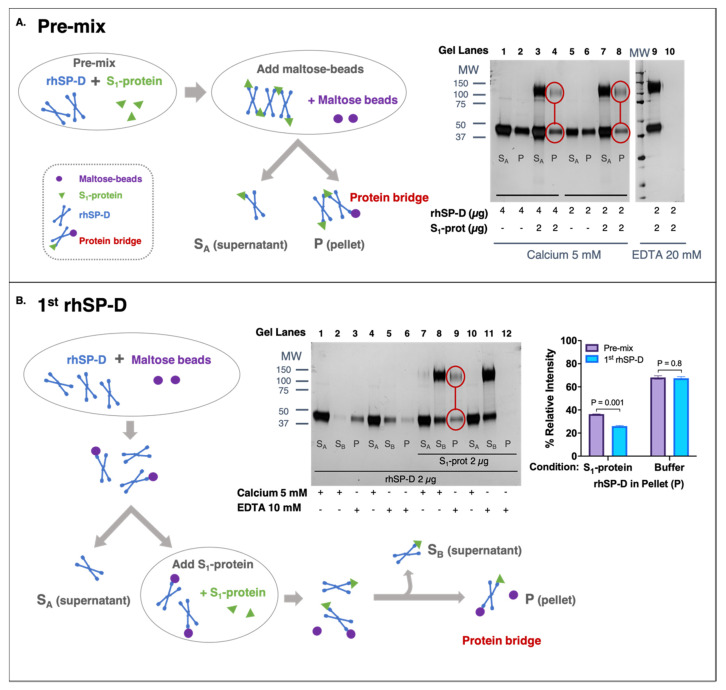
rhSP-D forms protein bridges with the spike (S)-protein of SARS-CoV-2. (**A**,**B**) The fractions obtained from supernatant (S_A_ and S_B_) and eluted fraction from pellet (P) were analyzed by SDS-PAGE and developed by silver-staining to detect S_1_-protein (migrates as 100–140 kDa) and rhSP-D (43 kDa). (**A**) Pre-mix approach: rhSP-D and S_1_-protein (Wuhan variant) were incubated before adding maltose-coated beads. The process is described in the schematic diagram. “S_A_” supernatant contained rhSP-D bound to S_1_-protein, which did not interact with the maltose-beads. The eluted fraction (“P”) comprised the rhSP-D that was bound to both S_1_-protein and maltose-beads, forming a protein bridge. Two concentrations of rhSP-D were tested 4 μg (lanes 1–4) and 2 μg (lanes 5–10) with 2 μg S_1_-protein (lanes 3, 4, 7, 8, 9, 10) or only buffer as control (lanes 1, 2, 5, 6). The experiment was performed in the presence of 5 mM calcium (lanes 1–8), except elution of the pellet (P), which was carried out with 20 mM EDTA; non-binding control was performed in the presence of 20 mM EDTA (lanes 9–10). (**B**) The 1st-rhSP-D approach: rhSP-D was first incubated with maltose beads. Then, the excess of rhSP-D was removed (supernatant “S_A_”) and S_1_-protein (Wuhan variant) was added. “S_B_” supernatant contained rhSP-D only bound to S_1_-protein, and the eluted fraction (P) contained rhSP-D that remained bound to the maltose beads and also to S_1_-protein, forming the protein bridges. Samples contained 2 μg of rhSP-D and 2 μg S_1_-protein (lanes 7–12) or buffer (lanes 1–6). Binding of rhSP-D to maltose beads was always performed in the presence of calcium (S_A_, lanes: 1, 4, 7, 10). Binding to S_1_-protein was performed at 5 mM calcium (S_B_, lanes: 2, 8) or 20 mM EDTA as non-binding control (S_B_, lanes: 5, 11); elution (P) was carried out with 20 mM EDTA (E, lanes: 3, 6, 9, 12). The bar graph shows the densitometry of the eluted (P) bands in the pre-mix VS 1st-rhSP-D approaches at 4 μg of rhSP-D in the presence of S_1_-protein or buffer; ANOVA with Sidak post-test was performed, error bars represent standard deviation, densitometry (*n* = 2).

To determine if rhSP-D could aggregate SARS-CoV-2, the ability of rhSP-D to link S-protein to a second molecule (maltose-coated beads) was examined. Results demonstrate that rhSP-D formed a protein bridge with S_1_-protein (Wuhan variant) (“P” in Figure 2A: lane 4, 8; Figure 2: lane 9) and maltose-coated beads.

The formation of protein bridges by rhSP-D was inhibited by EDTA and therefore calcium-dependent (Figure 2A: lane 10; Figure 2B: lane 12). Binding between S-protein and rhSP-D was also confirmed in this second assay because fraction “S_B_” only contained rhSP-D in the presence of S_1_-protein (Figure 2B, lane 2 VS lane 8). The addition of S_1_-protein to rhSP-D that was previously bound to maltose-coated beads showed that part of that rhSP-D shifted and preferentially bound to the S-protein (observed in “S_B_” fractions). To determine if rhSP-D could form an aggregate of multiple S-protein and rhSP-D molecules, the pre-mix and 1st-rhSP-D approaches were compared. The pre-mix approach (Figure 2A) should allow the formation of larger order S-protein and rhSP-D aggregates of multiple S-protein and rhSP-D molecules. In contrast, binding of rhSP-D to maltose beads first, followed by removal of unbound rhSP-D and then binding to S-protein, should be limited to single units of rhSP-D bound to S-protein and maltose (Figure 2B). The intensity of rhSP-D bands in the eluted (“P”) fraction, in the pre-mix approach, is stronger than their respective ones in the 1st-SP-D approach (Bar-graph of Figure 2B), which is consistent with the formation of larger-order aggregates. Collectively, these data demonstrate the existence of protein bridges facilitated by rhSP-D and suggest the aggregation of SARS-CoV-2 driven by rhSP-D.

### 3.4. ACE2 Receptor Does Not Interfere in the Interaction between S-Protein and rhSP-D

**Figure 3:**
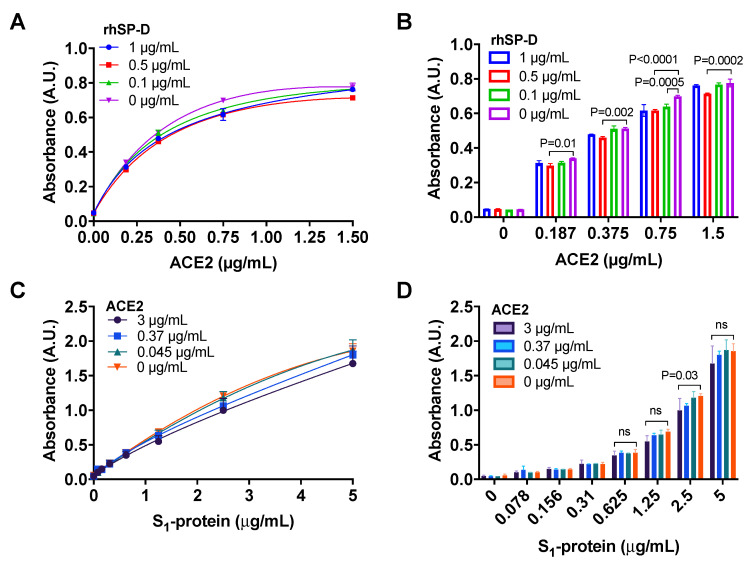
ACE2 does not interfere in the binding of rhSP-D to the spike-(S)-protein of SARS-CoV-2. (**A**,**B**) An ELISA assay was performed to determine if rhSP-D could decrease the binding of ACE2 to S_1_-protein (Wuhan variant). Binding of ACE2 to S_1_-protein immobilized on the wells was determined in the presence of different concentrations of rhSP-D 1 μg/mL (blue, circles), 0.5 μg/mL (red, squares) or 0.1μg/mL (green, upward triangles) and 0 μg/mL (pink, downward triangles) (positive control for the binding of ACE2 to S_1_-protein). rhSP-D promoted a slight decrease in the interaction between S_1_-protein and ACE2. The reduction induced by rhSP-D at 0.5 μg/mL was significant compared to the positive control (no rhSP-D: 0 μg/mL). (**C**,**D**) Binding of S_1_-protein to rhSP-D (immobilized on the wells) in the presence of different concentrations of ACE2: 3 μg/mL (purple, circles), 0.37 μg/mL (blue, squares) or 0.045 μg/mL (green, upward triangles) and 0 μg/mL (orange, downward triangles) (positive control for binding of S_1_-protein to rhSP-D) was determined by ELISA. There was only a discrete reduction in the binding of S_1_-protein to rhSP-D at the highest concentration of ACE2 tested (3 μg/mL). A–D, ANOVA with Tukey post-test, *p*-values in bar graphs when significant. *n* = 2, error bars represent standard deviation for duplicates.

A marginal, although significant, decrease in the binding of ACE2 to S_1_-protein (Wuhan variant) in the presence of 0.5 μg/mL rhSP-D compared to the control without rhSP-D (Figure 3A,B) was observed.

The results also demonstrated that the addition of ACE2 did not inhibit the binding of rhSP-D to S_1_-protein (Figure 3C,D) until a small decrease in binding was observed at the maximum concentration of ACE2 (3 μg/mL). Therefore, rhSP-D and ACE2 may bind to different regions of S_1_-protein, allowing the co-interaction of the three molecules.

3.5. rhSP-D Inhibits SARS-CoV-2 Replication in Host Cells

**Figure 4:**
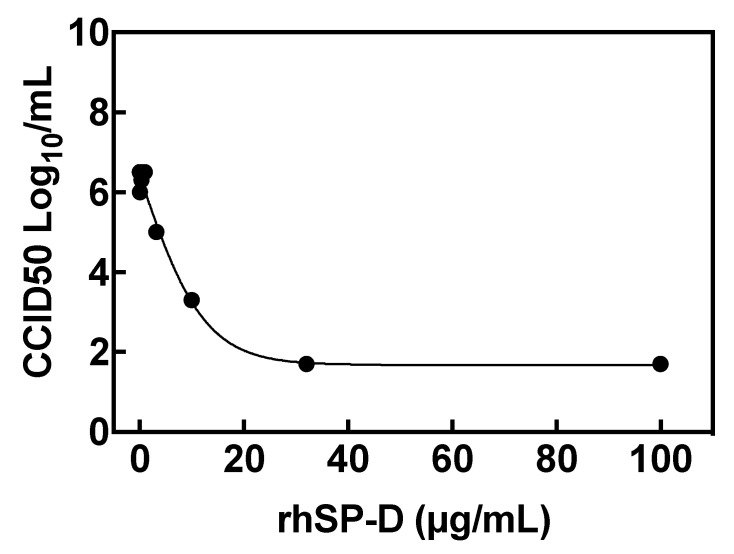
rhSP-D inhibits SARS-CoV-2 cell replication. Viral titers in cells after infection with SARS-CoV-2 and treatment with rhSP-D at different concentrations. The viral titer in the cell supernatant is reported as CCID50 (50% cell culture infectious dose). Individual data points represent the average of three replicates. The concentration of rhSP-D to inhibit viral replication by 90% (EC_90_) was 3.7 μg/mL.

The effect of rhSP-D on SARS-CoV-2 replication in host cells was tested in vitro with a viral replication assay in human epithelial Caco-2 cells. rhSP-D inhibited viral replication in a dose-dependent manner, with higher concentrations of rhSP-D leading to greater inhibition of viral replication, which was observed by measuring the virus titer in the cell supernatant at the different rhSP-D concentrations tested and reported as CCID50 (50% cell culture infectious dose) (Figure 4).

The concentration of rhSP-D to inhibit viral replication by 90% (EC_90_) was 3.7 μg/mL. Moreover, rhSP-D did not show any cell toxicity even at the highest rhSP-D tested (100 μg/mL) when compared to control (non-treated and non-infected) cells.

## 4. Discussion

This study shows evidence of the importance of SP-D in COVID-19 and suggests the potential of rhSP-D as a COVID-19 anti-viral therapy. Our data demonstrates that full-length rhSP-D binds to SARS-CoV-2 spike-protein, inhibits viral replication in host cells, and initiates viral aggregation which could result in a more effective clearance of the virus by phagocytic cells.

Several studies have demonstrated the clinical significance of SP-D activity. A positive correlation has been shown between survival rates to ARDS and higher levels of pulmonary SP-D at the beginning of the syndrome [24]. Herein, we have shown that COVID-19 patients exhibit a decreased concentration of pulmonary SP-D (median = 68.9 ng/mL) compared with the levels reported in the literature for healthy subjects, which range from 900–1300 ng/mL [32,33,34]. Specifically, in Winkler et al. the BALF was collected with a very similar method and SP-D levels quantified with the same commercial ELISA kit [34]. We did not find any correlations between BALF SP-D concentrations and the clinical parameters provided (BMI, age, days of intubation, and days of hospitalization). Unfortunately, we recognize a significant downfall in our study is that BALF samples from a control cohort could not be obtained due to the overwhelmed status of hospitals and the risk of exposure in non-COVID-19 intubated control patients during the COVID-19 pandemic. Besides, the number of samples collected was also limited due to the epidemic situation of hospitals in March–April of 2020. In addition, the difficulty to identify a good control subject/sample for this heterogenous intubated population with different associated comorbidities make obtaining appropriate control samples a challenging pitfall to overcome. Still, the findings in this study suggest low pulmonary levels of SP-D in COVID-19 patients, and, therefore, supplementation of these critical patients with exogenous SP-D to increase levels of SP-D in lungs could improve outcomes in these patients.

Pathogen recognition and binding to glycosylated determinants is the first and hallmark action of SP-D to opsonize infectious agents [15,16] and facilitate their fast clearance by phagocytic cells in the lungs [18,19,35,36]. SP-D has shown calcium-dependent binding to the S-protein of the previous SARS-CoV strain [37], and high glycosylation of the current SARS-CoV-2 S-protein has been confirmed and mapped, suggesting SARS-CoV-2 S-protein may be a target of SP-D [31]. Herein, we have demonstrated that rhSP-D binds to the antigen of different variants of the current SARS-CoV-2 (Figure 1) via a calcium-dependent process that mimics opsonization and the critical first step of clearance of SARS-CoV-2 by SP-D in vivo. A recent study has shown binding of a small fragment of SP-D (rfhSP-D) to the S-protein of SARS-CoV-2, although the interaction was reported to be calcium-independent [38]. Differences with our data might be attributed to the use of the rfhSP-D variant rather the full-length protein. Our results indicate that rhSP-D binding to S-protein is calcium-dependent, which is the normal binding mechanism for SP-D that has been demonstrated for SP-D binding to SARS-CoV [37] and to other viruses such as respiratory syncytial virus and influenza A virus [18,25]. Binding affinity of SP-D for the spike protein of the original variant from Wuhan was very similar to the variant emerged in U.K. (B.1.1.7.), South Africa (B.1.351), and Brazil (P.1) (Figure 1F,G), which remarks the potential of rhSP-D as an anti-viral for all the current circulating variants of the virus. Many factors determine the infectivity and severity of the disease produced by the virus; recognizing that limitation, it is tempting to speculate that the binding affinity of SP-D to the spike protein could be one of the factors that could influence the virulence of emerging new variants [10,14,39], which could be translated in the virus, bypassing the innate immune defense more easily with the right combination of mutated amino acids. In line with this, it has been published that the N501Y spike mutation enhances virus transmission [13] and we found decreased binding affinity of SP-D to the spike protein with this single mutation.

Binding of pathogens by SP-D leads to their aggregation, forming clusters where multiple viral molecules that are removed at once by phagocytic cells. The critical first step of aggregation is driven by the ability of SP-D to bind more than one virus and form a protein bridge linking multiple pathogens. We have shown that rhSP-D is able to form protein bridges between S-proteins (Figure 2). Although our experiments do not demonstrate aggregation of the native virus, they do demonstrate the critical first step of viral aggregation (i.e., binding) and the subsequent formation of the rhSP-D protein bridge. Moreover, it is likely that the presence of multiple spike-proteins on the surface of the intact virus will further facilitate viral aggregation and clearance.

We have shown that rhSP-D inhibits SARS-CoV-2 life cycle by inhibiting virus replication in cells with an EC_90_ of 3.7 μg/mL (Figure 4), which is physiologically relevant as concentrations of exogenous rhSP-D at 3.7 μg/mL can be easily achieved when rhSP-D is administered therapeutically [40]. A proposed mechanism to explain this inhibition could be a steric blockage on the interaction between the receptor binding domain within S-protein and ACE2 by the rhSP-D bound to the glycosylated S-protein, which could restrict the accessibility of key domains in the presence of the bound SP-D molecule. However, this effect was not evident when we performed the experiments with isolated S_1_-protein, ACE2 and rhSP-D (Figure 3), it is possible that steric blockage may still be observed when the conformation and position of the S-protein and ACE2 receptor are restrained on a virus envelope or cell membrane, respectively. A second mechanism to explain rhSP-D inhibition of virus replication, which could cooperate with the first mechanism proposed, would be the potential aggregation of SARS-CoV-2 induced by rhSP-D by reducing the number of viral molecules available to interact with the host cell, however, further experiments are needed to confirm this second hypothesis. Interestingly, it has been recently published that the trimeric fragment rfhSP-D containing only the carbohydrate-binding domain and neck regions of SP-D inhibited viral entry in cells using pseudotyped lentiviral particles expressing SARS-CoV-2 S1 protein [41]. One could speculate that the same or even stronger effect should be expected when using full length rhSP-D, testing this hypothesis in lung epithelial cells is very interesting and will be done as part of future work in this project. It would be also interesting to explore the endogenous binding of the spike protein to SP-D in the cells, perhaps using co-immunoprecipitation assays.

COVID-19 patients have shown a complex ARDS characterized by a cytokine storm of inflammation, thick mucus secretions in airways containing neutrophil extracellular traps (NETs), and extensive lung damage [5,6,7,42]. Importantly, SP-D has demonstrated antiinflammatory activities in lungs that occur independently of pathogen binding. SP-D mediates the inhibition of inflammation signaling through TLR-4, TLR-2, and SIRP-α, followed by a reduction of NF-κB activation [15,43,44,45]. Mice with deletion of the Sftpd gene have shown elevated oxygen radical release, neutrophil NETs, and production of the proinflammatory mediators when exposed to either viral or bacterial pathogens [20,22,23,46]. Therefore, these data indicate that the administration of rhSP-D may also decrease the exacerbated inflammatory response observed in COVID-19 patients.

In conclusion, we have shown that COVID-19 patients have low pulmonary levels of SP-D. Full length recombinant hSP-D binds the SARS-CoV-2 S-protein from different physiologically relevant variants and, importantly, inhibits the life cycle of the virus by inhibiting viral replication. SP-D can form protein bridges with S-protein, which represents the first step of viral aggregation that would enhance viral clearance from the lungs by phagocytic cells. In addition, SP-D has previously demonstrated antiinflammatory and lung-protective roles in several viral and bacterial infections. SP-D has strong potential to be a novel class of antiviral therapy that will target multiple stages of the SARS-CoV-2 infection.

## Figures and Tables

**Table 1:** Characteristics of patients, hospitalization and intubation time (days) at BAL sample collection.

Age	Sex	BMI	BALF SP-D	Collection of BAL Sample at		Comorbidities and/or Smoking
				Hospitalization Day	Intubation Day	
28	F	26.6	55	9	3	N
40	M	44.2	83	11	10	N
46	F	29.4	7	14	14	N
50	M	34.6	107	3	3	Y (HIV)
53	M	26.2	32	11	9	Y (smoker)
55	M	24.8	479	24	13	Y (smoker, CV, cancer)
60	M	-	752	4	3	Y (CV)
61	M	21.6	8	6	5	Y (cancer)
64	M	32.8	1145	6	6	Y (CV)
68	F	23.4	217	9	9	N
68	M	25.2	45	50	50	N
73	M	29.4	7	4	3	N

Sex: M (male), F (female). BMI (body mass index) above 30 considered obesity. The comorbidities that were screened: cardiovascular disease (CV), respiratory disease, immunosuppression, human immunodeficiency virus (HIV), diabetes mellitus type I and type II, and cancer; smoking was also reported; absence of any of these comorbidities and/or smoking is indicated as “N”, presence of them is indicated with “Y” indicating the comorbidity in brackets.

## Data Availability

The data presented in this study are available in this article and supplementary material.

## Supplementary Materials

The following are available online at https://www.mdpi.com/article/10.3390/biom11081114/s1, Supplemental Figure S1: the spike-(S)-protein of SARS-CoV-2 binds to rhSP-D. ELISA binding assays were performed coating the plate wells with with rhSP-D. The binding was determined under different conditions: 5 mM calcium (blue, circles), 200 mM maltose (red, squares) and 50 mM EDTA (green, upside triangles); additionally, wells were coated with bovine serum albumin (BSA) instead of rhSP-D to determine nonspecific binding and show that the binding between rhSP-D and S-protein was specific (grey, downside triangles). Maltose and BSA conditions were tested in presence of 5 mM calcium. *n* = 2, Error bars represent standard deviation of duplicates. The binding of S1-protein to rhSP-D was determined by anti-S1-protein-mFc ELISA. The S1-protein bound to rhSP-D in the presence of calcium was significantly different to the rhSP-D bound in the presence of EDTA (*p* = 0.002) or to BSA (*p* < 0.0001) (Kruskal-Wallis with Dunn’s post-test). Applying a one-binding site model the apparent Kd was 2.02 and the apparent Bmax was 0.81.

## References

[1] Zhu N., Zhang D., Wang W., Li X., Yang B., Song J., Zhao X., Huang B., Shi W., Lu R. (2020). A Novel Coronavirus from Patients with Pneumonia in China, 2019. N. Engl. J. Med..

[2] Walls A.C., Park Y.J., Tortorici M.A., Wall A., McGuire A.T., Veesler D. (2020). Structure, Function, and Antigenicity of the SARS-CoV-2 Spike Glycoprotein. Cell.

[3] Lan J., Ge J., Yu J., Shan S., Zhou H., Fan S., Zhang Q., Shi X., Wang Q., Zhang L. (2020). Structure of the SARS-CoV-2 spike receptor-binding domain bound to the ACE2 receptor. Nature.

[4] Shang J., Ye G., Shi K., Wan Y., Luo C., Aihara H., Geng Q., Auerbach A., Li F. (2020). Structural basis of receptor recognition by SARS-CoV-2. Nature.

[5] Chen N., Zhou M., Dong X., Qu J., Gong F., Han Y., Qiu Y., Wang J., Liu Y., Wei Y. (2020). Epidemiological and clinical characteristics of 99 cases of 2019 novel coronavirus pneumonia in Wuhan, China: A descriptive study. Lancet.

[6] Wang D., Hu B., Hu C., Zhu F., Liu X., Zhang J., Wang B., Xiang H., Cheng Z., Xiong Y. (2020). Clinical Characteristics of 138 Hospitalized Patients With 2019 Novel Coronavirus-Infected Pneumonia in Wuhan, China. JAMA.

[7] Arentz M., Yim E., Klaff L., Lokhandwala S., Riedo F.X., Chong M., Lee M. (2020). Characteristics and Outcomes of 21 Critically Ill Patients With COVID-19 in Washington State. JAMA.

[8] Beigel J.H., Tomashek K.M., Dodd L.E., Mehta A.K., Zingman B.S., Kalil A.C., Hohmann E., Chu H.Y., Luetkemeyer A., Kline S. (2020). Remdesivir for the Treatment of Covid-19—Preliminary Report. N. Engl. J. Med..

[9] Horby P., Lim W.S., Emberson J.R., Mafham M., Bell J.L., Linsell L., Staplin N., Brightling C., Ustianowski A., The RECOVERY Collaborative Group (2020). Dexamethasone in Hospitalized Patients with Covid-19—Preliminary Report. N. Engl. J. Med..

[10] Tegally H., Wilkinson E., Giovanetti M., Iranzadeh A., Fonseca V., Giandhari J., Doolabh D., Pillay S., San E.J., Msomi N. (2021). Emergence of a SARS-CoV-2 variant of concern with mutations in spike glycoprotein. Nature.

[11] Rambaut A.L.N., Pybus O., Barclay W., Barrett J., Carabelli A., Connor T., Peacock T., Robertson D.L., Volz E. (2020). Preliminary Genomic Characterisation of an Emergent SARS-CoV-2 Lineage in the UK Defined by a Novel Set of Spike Mutations. https://virological.org/t/preliminary-genomiccharacterisation-of-an-emergent-sars-cov-2-lineage-in-the-uk-defined-by-anovel-set-of-spike-mutations/563.

[12] Voloch C.M., da Silva Francisco R., de Almeida L.G.P., Cardoso C.C., Brustolini O.J., Gerber A.L., Guimaraes A.P.C., Mariani D., da Costa R.M., Ferreira O.C. (2021). Genomic characterization of a novel SARS-CoV-2 lineage from Rio de Janeiro, Brazil. J. Virol..

[13] Liu Y., Liu J., Plante K.S., Plante J.A., Xie X., Zhang X., Ku Z., An Z., Scharton D., Schindewolf C. (2021). The N501Y spike substitution enhances SARS-CoV-2 transmission. bioRxiv.

[14] Rees-Spear C., Muir L., Griffith S.A., Heaney J., Aldon Y., Snitselaar J.L., Thomas P., Graham C., Seow J., Lee N. (2021). The effect of spike mutations on SARS-CoV-2 neutralization. Cell Rep..

[15] Wright J.R. (2005). Immunoregulatory functions of surfactant proteins. Nat. Rev. Immunol..

[16] Kingma P.S., Whitsett J.A. (2006). In defense of the lung: Surfactant protein A and surfactant protein D. Curr. Opin. Pharm..

[17] Arroyo R., Echaide M., Moreno-Herrero F., Perez-Gil J., Kingma P.S. (2020). Functional characterization of the different oligomeric forms of human surfactant protein SP-D. Biochim. Biophys. Acta Proteins Proteom..

[18] LeVine A.M., Elliott J., Whitsett J.A., Srikiatkhachorn A., Crouch E., DeSilva N., Korfhagen T. (2004). Surfactant protein-d enhances phagocytosis and pulmonary clearance of respiratory syncytial virus. Am. J. Respir. Cell Mol. Biol..

[19] Ikegami M., Carter K., Bishop K., Yadav A., Masterjohn E., Brondyk W., Scheule R.K., Whitsett J.A. (2006). Intratracheal recombinant surfactant protein D prevents endotoxin shock in the newborn preterm lamb. Am. J. Respir. Crit. Care Med..

[20] Ikegami M., Scoville E.A., Grant S., Korfhagen T., Brondyk W., Scheule R.K., Whitsett J.A. (2007). Surfactant protein-D and surfactant inhibit endotoxin-induced pulmonary inflammation. Chest.

[21] Sato A., Whitsett J.A., Scheule R.K., Ikegami M. (2010). Surfactant protein-D inhibits lung inflammation caused by ventilation in premature newborn lambs. Am. J. Respir. Crit. Care Med..

[22] King B.A., Kingma P.S. (2011). Surfactant protein D deficiency increases lung injury during endotoxemia. Am. J. Respir. Cell Mol. Biol..

[23] Arroyo R., Khan M.A., Echaide M., Perez-Gil J., Palaniyar N. (2019). SP-D attenuates LPS-induced formation of human neutrophil extracellular traps (NETs), protecting pulmonary surfactant inactivation by NETs. Commun. Biol..

[24] Greene K.E., Wright J.R., Steinberg K.P., Ruzinski J.T., Caldwell E., Wong W.B., Hull W., Whitsett J.A., Akino T., Kuroki Y. (1999). Serial changes in surfactant-associated proteins in lung and serum before and after onset of ARDS. Am. J. Respir. Crit. Care Med..

[25] Hartshorn K., Chang D., Rust K., White M., Heuser J., Crouch E. (1996). Interactions of recombinant human pulmonary surfactant protein D and SP-D multimers with influenza A. Am. J. Physiol..

[26] Arroyo R., Martin-Gonzalez A., Echaide M., Jain A., Brondyk W.H., Rosenbaum J., Moreno-Herrero F., Perez-Gil J. (2018). Supramolecular Assembly of Human Pulmonary Surfactant Protein SP-D. J. Mol. Biol..

[27] Arroyo R., Echaide M., Wilmanowski R.M., Martin-Gonzalez A., Batllori E., Galindo A., Rosenbaum J.S., Moreno-Herrero F., Kingma P.S., Perez-Gil J. (2020). Structure and activity of human surfactant protein sp-d from different natural sources. Am. J. Physiol. Lung Cell Mol. Physiol..

[28] Pandolfi L., Fossali T., Frangipane V., Bozzini S., Morosini M., D’Amato M., Lettieri S., Urtis M., Di Toro A., Saracino L. (2020). Broncho-alveolar inflammation in COVID-19 patients: A correlation with clinical outcome. BMC Pulm. Med..

[29] Reed L.J., Muench H. (1938). A simple method of estimating fifty per cent endpoints12. Am. J. Epidemiol..

[30] Sorensen G.L., Husby S., Holmskov U. (2007). Surfactant protein A and surfactant protein D variation in pulmonary disease. Immunobiology.

[31] Watanabe Y., Allen J.D., Wrapp D., McLellan J.S., Crispin M. (2020). Site-specific glycan analysis of the SARS-CoV-2 spike. Science.

[32] Honda Y., Kuroki Y., Matsuura E., Nagae H., Takahashi H., Akino T., Abe S. (1995). Pulmonary surfactant protein D in sera and bronchoalveolar lavage fluids. Am. J. Respir. Crit. Care Med..

[33] Hermans C., Bernard A. (1999). Lung epithelium-specific proteins: Characteristics and potential applications as markers. Am. J. Respir. Crit. Care Med..

[34] Winkler C., Atochina-Vasserman E.N., Holz O., Beers M.F., Erpenbeck V.J., Krug N., Roepcke S., Lauer G., Elmlinger M., Hohlfeld J.M. (2011). Comprehensive characterisation of pulmonary and serum surfactant protein D in COPD. Respir. Res..

[35] Hartshorn K.L., Crouch E., White M.R., Colamussi M.L., Kakkanatt A., Tauber B., Shepherd V., Sastry K.N. (1998). Pulmonary surfactant proteins A and D enhance neutrophil uptake of bacteria. Am. J. Physiol..

[36] LeVine A.M., Whitsett J.A., Hartshorn K.L., Crouch E.C., Korfhagen T.R. (2001). Surfactant protein D enhances clearance of influenza A virus from the lung in vivo. J. Immunol..

[37] Leth-Larsen R., Zhong F., Chow V.T., Holmskov U., Lu J. (2007). The SARS coronavirus spike glycoprotein is selectively recognized by lung surfactant protein D and activates macrophages. Immunobiology.

[38] Madan T., Biswas B., Varghese P.M., Subedi R., Pandit H., Idicula-Thomas S., Kundu I., Rooge S., Agarwal R., Tripathi D.M. (2021). A Recombinant Fragment of Human Surfactant Protein D Binds Spike Protein and Inhibits Infectivity and Replication of SARS-CoV-2 in Clinical Samples. Am. J. Respir. Cell Mol. Biol..

[39] Zhou D., Dejnirattisai W., Supasa P., Liu C., Mentzer A.J., Ginn H.M., Zhao Y., Duyvesteyn H.M.E., Tuekprakhon A., Nutalai R. (2021). Evidence of escape of SARS-CoV-2 variant B.1.351 from natural and vaccine-induced sera. Cell.

[40] Arroyo R., Grant S.N., Gouwens K.R., Miller D.M., Kingma P.S. (2021). Evaluation of recombinant human SP-D in the rat premature lung model. Ann. Anat..

[41] Hsieh M.H., Beirag N., Murugaiah V., Chou Y.C., Kuo W.S., Kao H.F., Madan T., Kishore U., Wang J.Y. (2021). Human Surfactant Protein D Binds Spike Protein and Acts as an Entry Inhibitor of SARS-CoV-2 Pseudotyped Viral Particles. Front. Immunol..

[42] Zuo Y., Yalavarthi S., Shi H., Gockman K., Zuo M., Madison J.A., Blair C.N., Weber A., Barnes B.J., Egeblad M. (2020). Neutrophil extracellular traps in COVID-19. JCI Insight.

[43] Gardai S.J., Xiao Y.Q., Dickinson M., Nick J.A., Voelker D.R., Greene K.E., Henson P.M. (2003). By binding SIRPalpha or calreticulin/CD91, lung collectins act as dual function surveillance molecules to suppress or enhance inflammation. Cell.

[44] Ohya M., Nishitani C., Sano H., Yamada C., Mitsuzawa H., Shimizu T., Saito T., Smith K., Crouch E., Kuroki Y. (2006). Human pulmonary surfactant protein D binds the extracellular domains of Toll-like receptors 2 and 4 through the carbohydrate recognition domain by a mechanism different from its binding to phosphatidylinositol and lipopolysaccharide. Biochemistry.

[45] Yamazoe M., Nishitani C., Takahashi M., Katoh T., Ariki S., Shimizu T., Mitsuzawa H., Sawada K., Voelker D.R., Takahashi H. (2008). Pulmonary surfactant protein D inhibits lipopolysaccharide (LPS)-induced inflammatory cell responses by altering LPS binding to its receptors. J. Biol. Chem..

[46] Kingma P.S., Zhang L., Ikegami M., Hartshorn K., McCormack F.X., Whitsett J.A. (2006). Correction of pulmonary abnormalities in Sftpd−/− mice requires the collagenous domain of surfactant protein D. J. Biol. Chem..

