# Supplementary material for: Full-Length Recombinant hSP-D Binds and Inhibits SARS-CoV-2
Source: Biomolecules. 2021 Jul 28;11(8):1114. doi: 10.3390/biom11081114 (PMC8393632; doi:10.3390/biom11081114)
Supplement: Supplementary file 1 [file biomolecules-11-01114-s001.zip › biomolecules-1253024-supplementary.pdf]

Supplemental Figure E1

Methods for Supplemental Figure E1:

A second ELISA assay was developed, in which the wells were coated with rhSP-D at 5 µg/mL instead of S_1-protein. In this case, samples of serial diluted S_1-protein with a mouse Fc tag (mFc) (from 10 µg/mL to 9.8 ng/mL) were added to the wells. Bound S_1-protein was detected with an anti-mouse IgG horseradish peroxidase (HRP)-conjugated antibody (1:5000) (#7076, Cell Signaling). The analysis of the data in this case was performed subtracting the absorbance of the blank at each specific condition (calcium, maltose, EDTA or BSA) from the related samples.

Analysis of the binding isotherms was performed with GraphPad Prism 8, considering total binding and one site to determine the apparent dissociation constant (kd).

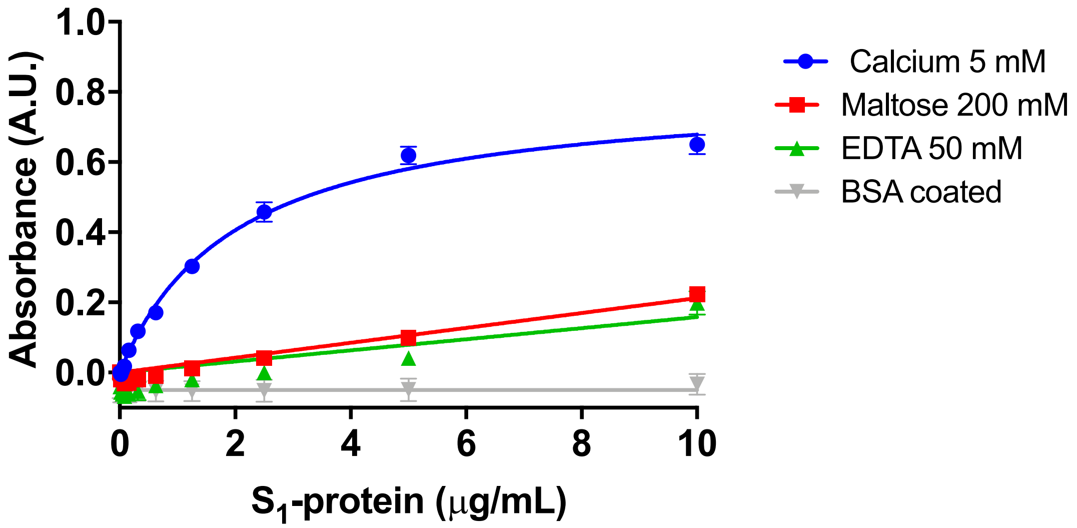

Supplemental Figure S1: the spike-(S)-protein of SARS-CoV-2 binds to rhSP-D. ELISA binding assays were performed coating the plate wells with with rhSP-D. The binding was determined under different conditions: 5 mM calcium (blue, circles), 200

mM maltose (red, squares) and 50 mM EDTA (green, upside triangles); additionally, wells were coated with bovine serum albumin (BSA) instead of rhSP-D to determine nonspecific binding and show that the binding between rhSP-D and S-protein was specific (grey, downside triangles). Maltose and BSA conditions were tested in presence of 5 mM calcium. N=2, Error bars represent standard deviation of duplicates. The binding of S_1-protein to rhSP-D was determined by anti-S_1-protein-mFc ELISA. The S_1-protein bound to rhSP-D in the presence of calcium was significantly different to the rhSP-D bound in the presence of EDTA ($p=0.002$) or to BSA ($p<0.0001$) (Kruskal-Wallis with Dunn's post-test). Applying a one-binding site model the apparent Kd was 2.02 and the apparent B_{max} was 0.81.
